# Assessment of a psychiatric intervention at community level for people who inject drugs in a low-middle income country: the DRIVE-Mind cohort study in Hai Phong, Viet Nam

**DOI:** 10.1016/j.lanwpc.2021.100337

**Published:** 2021-12-13

**Authors:** Laurent Michel, Sao Mai Le, Giang Hoang Thi, Philippe Trouiller, Huong Duong Thi, Oanh Khuat Thi Hai, Khue Pham Minh, Roselyne Vallo, Delphine Rapoud, Catherine Quillet, Thuy Linh Nguyen, Quang Duc Nguyen, Tuyet Thanh NhamThi, Jonathan Feelemyer, Vinh Vu Hai, Jean-Pierre Moles, Hong Quang Doan, Didier Laureillard, Don C. Des Jarlais, Nicolas Nagot, DRIVE Study Team

**Affiliations:** a CESP Inserm UMRS 1018, Paris Saclay University, Pierre Nicole Center, French Red Cross, 27 rue Pierre Nicole, 75005 Paris, France; b Hai Phong University of Medicine and Pharmacy, 72A Nguyễn Bỉnh Khiêm, Đằng Giang, Ngô Quyền, Hai Phong, Vietnam; c Supporting Community Development Initiatives, 240 Mai Anh Tuan, Thanh Cong Ward, Ba Dinh District, Hanoi, Vietnam; d Pathogenesis and control of chronic and emerging infections, University of Montpellier, Inserm, Etablissement Français du Sang, University of Antilles, 60 Rue de Navacelles, 34394 Montpellier, France; e New York University, College of Global Public Health, 665 Broadway Suite 800, NY 10013 New York, USA; f Dept of Infectious and tropical diseases, Viet Tiep Hospital, Số 1 Đường nhà thương - Quận Lê Chân, Hai Phong, Vietnam; g Infectious Diseases Department, Caremeau University Hospital, Place du Professeur Robert Debré, 30029 Nîmes, France

**Keywords:** ANRS, French Agency for Research on AIDS and Viral Hepatitis, CBO, community-based organization, CGI, clinical global impression scale, DRIVE, Drug-Related Infections in ViEtnam, EQ5D5L, 5 levels/5 dimensions EuroQol instrument, HIV, human immunodeficiency virus, LMICs, low-middle income countries, MINI, MINI international neuropsychiatric interview, MMT, methadone maintenance treatment, NIDA, National Institute on Drug Abuse, PHQ, patient health questionnaire, PWID, people who inject drugs, RDS, respondent driven sampling, SCDI, Supporting Community Development Initiatives, VND, Vietnamese dong

## Abstract

**Background:**

Access to psychiatric care for people who inject drugs (PWID) is limited/absent and stigmatized in most low-middle-income countries (LMICs). Innovative interventions are needed. We aimed to describe and assess the impact of a community-based psychiatric intervention among PWID in Hai Phong, Vietnam

**Methods:**

In a cohort study with one year psychiatric follow-up, PWID diagnosed with a psychotic disorder, a major depressive episode, or suicide risk, were recruited from the wider Drug-Related Infections in ViEtnam (DRIVE) project in the city of Hai Phong. The community-based psychiatric intervention included specialized follow-up (free consultations with psychiatrists, free medication, referral to mental health department for hospitalization when necessary) and support from community-based organisations (case management, harm reduction, administrative support, linkage to HIV care, methadone maintenance treatment and mental health support). The main outcome was reduction/remission of symptoms. Access to and retention in psychiatric care, quality-of-life and stigmatization were also measured pre and post-intervention.

**Findings:**

Among the 1212 participants screened from March to May 2019, 271 met the inclusion criteria, 233 (86.3%) accepted the intervention and 170 completed the follow-up (72.9%). At inclusion, 80.6% were diagnosed with current depression, 44.7% with psychotic disorder and 42.4% with suicide risk. After a one-year follow-up, these proportions dropped to 15.9%, 21.8%, and 22.9% respectively. Quality-of-life and perceived stigma related to mental health were also significantly improved, while drug use decreased only marginally.

**Interpretation:**

Community-based psychiatric interventions are both feasible and efficient in the Vietnamese context. Similar interventions should be implemented and evaluated in other, different LMICs.

**Funding:**

: This work was supported by grants from NIDA (US) (#DA041978) and ANRS (France) (#13353). The funding agencies had no role in designing the research, data analyses, or preparation of the report.

**Research in context**

**Evidence before this study**

Access to psychiatric care is often poor in low-middle income countries (LMICs), particularly for subjects with co-occurring psychiatric and substance use disorders, as a result of limited psychiatric resources and stigma. Innovative and alternative care organizations, including community-based and peer-supported interventions have been suggested as a relevant response, but are rarely reported.

**Added value of this study**

To our knowledge, this is the first study to describe a comprehensive community-based psychiatric intervention for people who inject drugs with co-occurring psychiatric and substance use disorders in LMICs. This intervention, involving different community-based organizations in the city of Hai Phong (Vietnam) and the mental health department of the University Hospital, proved its ability to initiate and maintain in care people who inject heroin, often smoke methamphetamines, and are diagnosed with a concurrent major depressive episode, a psychotic disorder or an on-going suicide risk. Significant clinical and quality-of-life improvements, as well as reduced stigma, were observed over a 12-month cohort follow-up period. This study clearly shows that, with only limited resources, an innovative and efficient peer-supported psychiatric intervention can be developed. This study drafts a model of what could be an alternative psychiatric intervention for key/hard-to-reach populations suffering from mental health disorders in LMICs. For ethical reasons, it was impossible to include a control group in the study design, as all participants in an earlier pilot study failed to initiate care in the classic mental health care system.

**Implications of all the available evidence**

Appropriate training for peer-support workers, steady salary and an alliance with psychiatrists were key components of the intervention. Peer involvement seems more fruitful than a classic intervention for little-known/hard-to-reach populations suffering from mental health problems in LMICs. It requires recognition of their status with acceptable salaries and involvement in the various stages of construction of the intervention. Alliance with mental health professionals implies recognition by these professionals of the experiential knowledge of peers on drug use, and also mental disorders. The cost, cost-effectiveness, and conditions for the sustainability of interventions of this sort still need to be assessed. These elements will be crucial for advocacy with health authorities. Lastly, our results need to be replicated in other hard-to-reach populations, such as men who have sex with men or transgender woman using drugs, young people who use drugs or sex workers with addictive behaviours.

Alt-text: Unlabelled box

## Introduction

Psychiatric disorders are very common among people who use drugs, reaching 40% among people who inject drugs (PWID), mainly mood, anxiety and personality disorders, but they vary considerably according to the drugs.^1^, ^2^, ^3^, ^4^ The use of stimulants is frequently associated with psychiatric complications, and in particular methamphetamines, with sometimes long-lasting psychotic disorders.^1^^,^^5^^,^^6^ People suffering from mental health disorders are more likely to engage in high-risk behaviours, to have late access methadone treatment when needed, and to have poorer health-seeking behaviour.^7^, ^8^, ^9^, ^10^, ^11^, ^12^, ^13^

Therefore, integrating mental health care into a multidisciplinary, holistic approach for key populations has become a major recommendation but faces considerable structural and financial obstacles in many parts of the world.^7^^,^^14^, ^15^, ^16^ In low and middle-income countries (LMICs), psychiatric resources are particularly limited and mental health care is stigmatized, adding to the discrimination, stigma and violence that these populations already have to face.^17^^,^^18^ To overcome these barriers, there is a need to develop innovative, alternative interventions, particularly community-based interventions, based on skill transfer and task shifting, involving peer-support.^14^^,^^19^, ^20^, ^21^, ^22^

Psychiatric resources in Vietnam are sparse, mental health literacy is low and mental disorders are associated with stigmatization and discrimination.^23^, ^24^, ^25^, ^26^, ^27^ In 2014, the number of psychiatrists in Vietnam was around 0.9 per 100,000 inhabitants, which is over 15 times and 23 times lower than in Australia (13.5 per 100,000 inhabitants) and in Japan (20.1 per 100,000 inhabitants) respectively.^28^^,^^29^ The numbers of nurses and psychologists in psychiatric settings are respectively 31 times lower (2.92 vs. 90.9 per 100, 000 inhabitants) and more than 1000 times lower (0.09 vs. 103.0 per 100,000 inhabitants) than in Australia.^28^^,^^29^ A pilot study conducted in 2016 among PWID in Hai Phong, the third largest city in the country and main port of North Vietnam, showed that none of the 48 subjects identified with a psychiatric disorder in the community, even those with the most severe diagnosis, were successfully referred to psychiatric clinics.^30^ The main reasons identified were fees associated with mental health care, lack of awareness and stigma, despite the fact that Hai Phong city had an estimated population of 5,000 PWID in 2016 and is known to possess a high level of harm reduction services cover.^30^, ^31^, ^32^

The objective of this study was to assess the impact of a psychiatric intervention at community level on the evolution of symptoms among PWID in Hai Phong, Vietnam. Access to and retention in care for psychiatric conditions were also evaluated.

## Materials and methods

### The DRIVE project (Drug use and Infections in ViEtnam)

Starting in 2016, a research program (DRIVE project) was implemented among PWID in Hai Phong, aiming to end the HIV epidemic in this population through a combined community-based intervention including repeated HIV testing, linkage to care (antiretroviral therapy, methadone, mental health), harm reduction and administrative support.^33^ PWID were recruited during three successive annual respondent-driven sampling (RDS) surveys (October to December 2016, 2017 and 2018) and some were invited to participate in a cohort for long-term evaluation of the intervention via biannual visits (sampling methods are detailed elsewhere).^33^^,^^34^ Criteria for inclusion in the different RDS surveys were: age 18 or over, self-reported current drug injection, confirmed by presence of recent skin injection marks and positive urinalysis for heroin and/or methamphetamine, residence in Hai Phong, and ability to provide informed consent.

Before the initiation of the DRIVE-Mind project, a regular, free psychiatric consultation was made available for all cohort participants in the community-based organization (CBO) offices where the DRIVE intervention was implemented.

### DRIVE-Mind project

#### Recruitment procedure – diagnosis process

The selection of subjects eligible for the intervention entailed the following procedure: first, a brief psychiatric screening was performed by CBO staff for all participants in the DRIVE cohort in the course of a follow-up visit. CBO staff were trained to submit a brief questionnaire^35^ including 4 questions on anxiety and depression (translated version of the Patient Health Questionnaire - PHQ4), 2 questions exploring suicide risk (past history of suicide attempt and current suicidal ideation) and 3 questions on psychotic symptoms, extracted and adapted from the MINI international neuropsychiatric interview, exploring lifetime persecutory ideas, auditory hallucinations and mind reading (which are all symptoms found to be particularly frequent among regular methamphetamine users in a preliminary study, data not shown).^36^, ^37^, ^38^ All participants screened positive for a potential psychiatric disorder (scoring 2 or more on the PHQ 4 or positive answers to any question on suicide or psychotic symptoms) were referred to a psychiatrist for further evaluation during the same session in CBO offices (see supplementary material for information on the tool used for screening).

**Figure 1:**
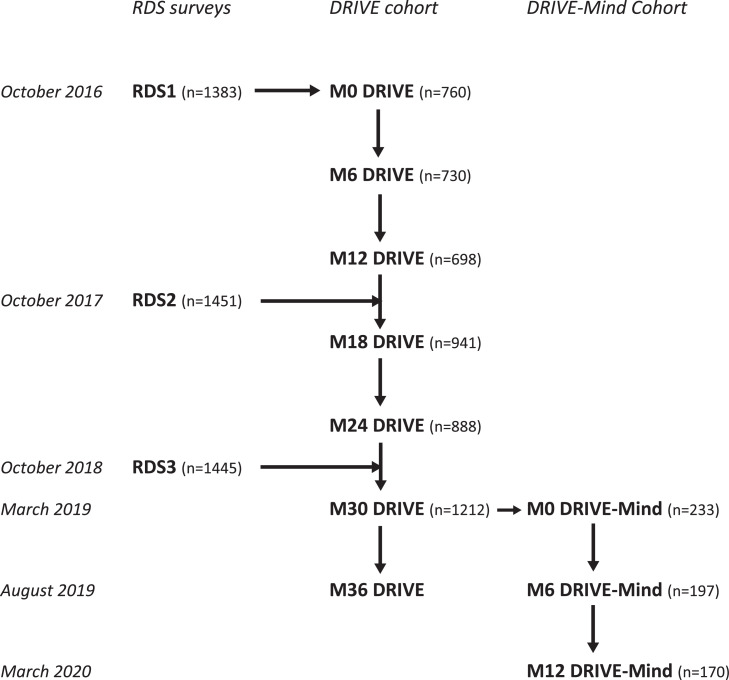
Overview of the DRIVE Project.

Trained psychiatrists from Hai Phong school of Medicine and Pharmacy administered the Mini-International Neuropsychiatric Interview (MINI 5.0.0) and provided their clinical input on the MINI diagnosis.^38^ Inclusion was also planned for participants with a negative MINI interview but exhibiting a significant isolated psychiatric symptom requiring an intervention (mainly severe anxiety, manic symptoms, or severe sleep disorder). DRIVE-Mind cohort participants were recruited from March 2019 to May 2019 during the month 30 visit in the DRIVE cohort (Figure 1). PWID eligible for recruitment in the DRIVE-Mind cohort were proposed a one-year follow-up, which included a visit at 6 months and a final visit at the end of the project (12 months).

### Outcomes and methods of measurement

The primary outcome was the evolution of psychiatric symptoms, measured by (i) MINI assessments followed by a clinical assessment and (ii) the Clinical Global Impression scale score (CGI).^39^ The MINI evaluation took place at M0, M6 and M12, and only the modules focusing on major depressive disorder, psychotic disorder and suicide risk were submitted to the participants. Two versions of the CGI scale were used: at baseline the CGI Severity scale (CGI-S) to assess the initial severity of the illness; at M6 and M12 visits the CGI Improvement scale (CGI-I) to evaluate the improvement in clinical situation compared to cohort initiation. Secondary outcomes were: the proportion of participants agreeing to initiate care among those identified with significant psychiatric symptoms at inclusion (accepting medical treatment/follow-up visits), the proportion of individuals still in follow-up at the end of the study (retention in psychiatric care), quality-of-life measured at M0, M6 and M12 via the 5-level and 5 dimensions EuroQol instrument (EQ5D5L), and lastly stigma associated with the psychiatric condition (investigated at M0 and M12 via the following question: “Do you feel ashamed to be diagnosed with a mental health problem?”).^40^ The EQ5D5L instrument is a standardized measure of health status with 5 components (anxiety, pain/discomfort, mobility, self-care, usual activities) and a score for perceived health where the participant is asked to score current health on a scale from 0 (worst health imaginable) to 100 (best health imaginable).

At each visit, data on drug use, sexual behaviours and use of drug-related services were collected in face-to-face structured interviews by trained CBO staff.

Urine samples were tested for heroin/morphine, methamphetamine, cannabis and methadone using Drug-screen Multi 7A carte (Nal von Minden, Germany) at each visit. HIV-negative participants were tested for HIV at each visit.

### Intervention

#### Psychiatric intervention

All psychiatric interventions took place in two different houses rented by CBOs with support of the non-government organization SCDI (Supporting Community Development Initiatives) which provides support throughout Vietnam for such initiatives.

#### Psychiatrists

Two psychiatrists from the mental health department were involved in the assessment and follow-up (support, therapeutic education, prescription) of the PWID included in the cohort. The frequency of appointments with participants for clinical care follow-up was scheduled according to the clinical situation of each PWID and varied from once a week to once a month.

#### Medication

Two antidepressants (sertraline and mirtazapin) and three antipsychotics (risperidone, olanzapine, sulpiride) were available and issued free by the psychiatrists at the end of consultations. Due to very restrictive regulation in Viet Nam, benzodiazepines were not used.

When needed, participants could be hospitalized in the mental health department and associated fees paid.

#### Community-based organizations

Seven community/peer support groups in Hai Phong (Friendship Arms, Light House, Sunrise, Lotus, An Duong Sun (for people who use drugs), Virgin Flowers (for female sex worker), and White Sands (for Men who have sex with men)) participated as full partners in developing the study procedures and provided expert knowledge of the local PWID situation. They were all former drug injectors or people affected by drugs and were involved in all steps of the RDS surveys, the DRIVE cohort and the DRIVE-Mind cohort and were in charge of around 20 peers each from the DRIVE cohort.

Training on psychiatric disorders and care was conducted in different sessions led by international experts (psychiatrists) and psychiatrists from the mental health department with support from SCDI staff familiar with these topics. It included formal training on mental health and relationships with drug use and risk behaviours, open discussions on care organization, and specific training on questionnaires used in follow-up visits. Flyers on methamphetamine use, often associated with psychiatric complications, and on mental health were designed together with CBO members to improve communication and introduce mental health concerns with their peers (see supplementary materials).

A limited number of CBO members (12 members) received additional training in Hai Phong or at National level with support from Hai Phongs' Medical University and SCDI on motivational interviewing, psychosocial interventions, drugs and drug interactions with mental health, in order to develop group intervention for their peers and families. The CBO tasks are listed in Table 1.

**Table 1:** CBO and psychiatrist tasks during the intervention.

CBO tasks related to mental health	- Individual and group information, education and communication on mental health, mental disorders, their treatments, side effects of the treatments and time to action, adherence to treatment - Distribution of flyers on harm reduction for methamphetamine users (including psychiatric consequences) and on mental health for peers and their family - Recall appointments with psychiatrists and payment for transportation fees - Information to psychiatrists in case of unusual events or worrying situation - Offer of closer follow-up for subjects signalled by the psychiatrist and when possible contact the family - Collection of information about participants lost of follow-up or in case of poor adherence - Referral of severe cases to hospital and payment for hospitalization fees when necessary - Meetings with family to inform, support and educate when necessary - Contact between family and doctors.
Other CBO tasks	- Linkage to HIV care and MMT - Administrative support (health insurance, identity card, resident card) - Harm reduction intervention (counselling, clean needles-syringes, condoms) - Collection of data on drug use, sexual behaviors and use of drug-related facilities during face-to-face structured interviews
Psychiatrists from the mental health department	- Free psychiatric consultations on CBO site - Free prescription on CBO site - Free delivery of treatment by psychiatrists on CBO site - Coordination of the follow-up

The salary for their intervention was 3,300,00 VND (143 US dollars) per month.

The purpose of the DRIVE-Mind project was thus to assess the ability of this finalized psychiatric community-based intervention to initiate care for people never exposed to any intervention, to maintain in care those already in care and overall to improve the clinical situation of PWID with co-occurring psychiatric and substance use disorders.

#### Data analysis

The first stage of the analysis was to compare characteristics of those who completed all visits and those who did not, using bivariate analysis. Then, among those who completed all visits, we compared psychiatric, drug use, and HIV outcomes at baseline (M0) and 12 months (M12) using paired t-test for continuous variables and McNemar test for categorical variables.

For continuous data, means, standard deviations, medians, interquartile ranges, and ranges values are given. For categorical data, absolute numbers and percentages are given, and 95% binomial proportion confidence intervals (CI) were calculated; the exact method was used when appropriate. These analyses were performed with R software version 4.0.2 (R Core Team, 2020). The threshold for statistical significance was set at p <0.05.

#### Ethical considerations

The research protocol was approved by the Institutional Ethics Committees of the Hai Phong Medicine and Pharmacy Faculty in Vietnam, the Icahn School of Medicine at Mount Sinai (New York city, USA) and the New York University School of Medicine (USA). Individual written informed consent was obtained from all participants prior to participation in each RDS and in the cohort study.

#### Role of the funding source

The funder of the study had no role in study design, data collection, data analysis, data interpretation, or writing of the report. The corresponding authors had full access to all the data in the study and had final responsibility for the decision to submit for publication.

## Results

In all, 1212 DRIVE participants were screened for a psychiatric disorder at the DRIVE-Mind M0 visit, 271 were diagnosed with a major depressive episode, a psychotic disorder, suicide risk or significant symptoms (severe sleep disturbances or anxiety symptoms, manic symptoms) requiring a psychiatric intervention, but only 233 signed their consent. Their mean age was 44 (standard deviation= 9), 22 (9%) were women, 77 (33%) were living with a partner, 143 (61%) had a health insurance, 13 (6%) had been homeless in the last 6 months, 166 (71%) had injected heroin in the last 6 months and 106 (46%) had inhaled methamphetamines in the last 30 days.

Among the 233 participants in the cohort, 131 (56%) had already received psychiatric medication as part of the DRIVE-project in the last 24 months, but only 18 (8%) reported still taking treatment on DRIVE-Mind cohort initiation.

**Figure 2:**
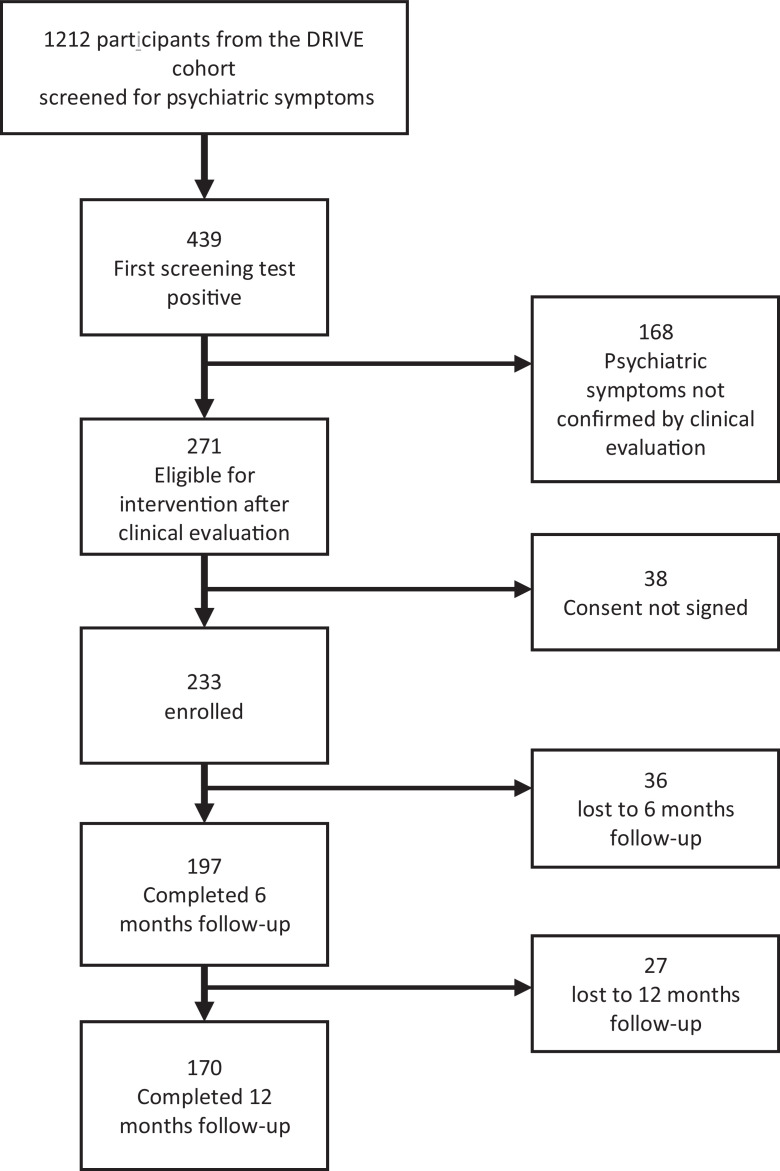
Flow chart for the DRIVE-Mind Cohort.

In the 12-month follow-up period, 197 (85%) participants returned for the M6 visit and 170 (73%) for the M12 visit (77% if we exclude the 12 participants who died, none from suicide, 3 from overdose) (see figure 2).

The acceptance rate for this intervention was 86% (233/271). Nearly 85% (32/38) of those who did not sign the consent form were free from current depressive or psychotic disorder. Their eligibility was based almost exclusively on the presence of a history of suicide attempt and their psychiatric symptomatology was mostly normal to mild according to the CGI Severity scale at cohort initiation. At baseline, medication indication was established for all 233 participants, who all accepted the prescribed medication.

In all, 63/233 participants were lost to follow-up. Among them, 12 died, 22 were incarcerated (13 for a drug-related offence, 3 for theft, 7 without information) and 6 were referred by the authorities to a compulsory treatment centre. Comparing data collected at cohort initiation, those lost to follow-up more frequently presented multiple psychiatric disorders at baseline and more severe illness according to the CGI Severity scale score than participants who completed the last visit (17.2% of subjects scored severely or extremely ill vs. 8.9%) (Table 2). They were slightly younger than those who completed the M12 visit (mean age= 41.8 vs. 44.2 years, p = 0.07). They were significantly less likely to have health insurance coverage (45.3% vs. 67.5 %, p= 0.003) and had a significantly greater likelihood of incarceration (23.4% vs. 9.5%, p= 0.01), indicating a more unstable social situation. Logically, they were significantly less likely to be taking methadone (39.1% vs. 65.7%, p<0.001). Although more patients lost to follow-up reported drug use (excluding alcohol), the differences between groups never reached statistical significance.

**Table 2:** Participant characteristics, lost-to-follow-up vs follow-up completed, comparison M0 vs. M12.

	Lost to follow-up	Follow-up completed (N = 170)			
	(N= 63)	M0	M6⁎⁎⁎	M12	*test*⁎⁎*(M0 vs. M12)*
**Age (mean (SD))**	41.83 (8.16)	44.17 (8.94)			*-*
**Gender (%)**					
Female	7 (11.1)	15 (8.8)			
Male	56 (88.9)	154 (90.6)			
Transgender	0 (0.0)	1 (0.6)			
**Marital status (%)**					*-*
single	17 (27.0)	57 (33.5)			
legally married	16 (25.4)	47 (27.6)			
living maritally	4 (6.3)	10 (5.9)			
separated	24 (38.1)	55 (32.4)			
widowed	2 (3.2)	1 (0.6)			
**Having a health insurance (%)**	28 (44.4)*	115 (67.6)	124 (73.4)	133 (78.2)	*0.01*
**Arrested in the last 6 months (%)**	15 (23.8)*	16 (9.4)	13 (7.7)	4 (2.4)	*0.01*
**Current methadone treatment (%)**	25 (39.7)*	111 (65.3)	114 (67.1)	115 (67.6)	*0.556*
**Heroin injection in the last 6 months (%)**	48 (76.2)	118 (69.4)	102 (60.4)	104 (61.2)	*0.045*
**Heroin injection: number of days in the last 30 days (median [IQR])**	20 [5-30]	22 [3-30]	20 [4-30]	20 [3.75-30]	*0.759*
**Shared at least once a needle in the last 6 months (%)**	2 (3.1)	4 (2.4)	0 (0)	0 (0)	*0.134*
**Shared/divided using a needle in the last 6 months (%)**	1 (1.6)	1 (0.8)	0 (0)	1 (1)	*1*
**Shared water in the last 6 months (%)**	1 (1.6)	3 (2.5)	1 (1)	2 (1.9)	*1*
**Meth use in the last 6 months (%)**	34 (54.0)	72 (42.4)	57 (33.7)	81 (47.6)	*0.188*
**Meth use: number of days in the last 30 days (median [IQR])**	5.5 [3-13.75]	6 [2-10]	5 [2-10]	4 [2-10]	*0.881*
**Regular methamphetamine use (at least 4 times in the last 30 days)**	23 (36.5)	43 (25.3)	33 (19.5)	46 (27.1)	*0.728*
**Alcohol use disorder (positive Audit-C score)**	15 (23.8)	45 (26.5)	37 (21.9)	32 (18.8)	*0.016*
**Regular polysubstance use**^a^**(%)**					*0.183*
0	19 (30.2)	65 (38.2)	78 (46.2)	75 (44.1)	
1	30 (47.6)	77 (45.3)	74 (43.8)	70 (41.2)	
2	11 (17.5)	23 (13.5)	14 (8.3)	22 (12.9)	
3	3 (4.8)	5 (2.9)	3 (1.8)	3 (1.8)	
**Do you feel ashamed of being diagnosed with a mental health problem? (%)**	29 (46.0)	93 (54.7)	42 (25)	28 (16.5)	*<0.001*
**Current major depressive episode^b^ (%)**	55 (87.3)	137 (80.6)	61 (35.9)	27 (15.9)	*<0.001*
**Current psychotic disorder**^b^**(%)**	36 (57.1)	76 (44.7)	53 (31.2)	37 (21.8)	*<0.001*
**Suicide risk (low, intermediate or high)^b^ (%)**	33 (52.4)	72 (42.4)	46 (27.2)	39 (22.9)	*<0.001*
**Number of psychiatric disorders**^c^**(%)**					*<0.001*
0	3 (4.8)	10 (5.9)		97 (57.1)	
1	16 (25.0)	65 (38.5)		49 (28.8)	
2	24 (37.5)	65 (38.5)		18 (10.6)	
3	20 (31.2)	30 (17.8)		6 (3.5)	
**Known HIV seropositivity**	23 (36.5)	73 (42.9)		73 (42.9)	*1*

a ^Among heroin,^^methamphetamine and alcohol^

b ^Based on the MINI^

c ^One disorder or more among the following: major depressive episode psychotic disorder suicide risk score > 0)^

⁎ ^significant difference between those lost to follow-up and M0 data for those who completed follow-up (p<0.05)^

⁎⁎ ^paired t-test for continuous variables and Mc Nemar test for categorical variables comparing M0 vs M12^

⁎⁎⁎ ^missing data for one subject only for M6 time-point meth: methamphetamine^

Analysis among those who completed follow-up (n=170, see Table 2)

### Psychiatric disorders

Among the 170 participants who completed the last visit, 80.6% were diagnosed with a current major depressive episode at inclusion, 44.7% presented a psychotic disorder, and suicide risk was identified for 42.4% (38.5% presented 2 conditions and 17.8% three of these conditions). Overall, 94.1 % of the participants presented at least one of the three above-mentioned psychiatric conditions, and approximately 6% of the subjects presented severe anxiety symptoms or sleep disorders. After 12 months of intervention, we observed a remarkable decrease for the two main disorders studied and suicide risk. The most significant improvement at 12 months was seen in depressive disorders (only 16% of participants had current major depressive disorder according to the MINI criteria, compared to 81% at inclusion, p<0.001), followed by psychotic disorders (22% vs. 45%, p<0.001) and suicide risk (23% vs. 42%, p<0.001). The proportion of participants with at least one of the above-mentioned three psychiatric conditions fell from 94.1% to 42.9% (p<0.001). Only three participants were hospitalized in a psychiatric department in the course of the one-year period. All presented a psychotic disorder and a suicide risk, and one was also diagnosed with major depressive disorder. One of them dropped out before the final visit. The CGI scale showed a clear improvement for 75% of the participants by the sixth month, and 79% by the twelfth month (Table 3). Minimum to no change was observed for 20% of the participants after twelve months. If we consider loss-to-follow-up as failure to improve psychiatric symptoms, 58% of the participants showed a clear improvement in their overall psychiatric condition after twelve months. (69% after excluding those who died, were incarcerated or were referred to compulsory treatment centre).

**Table 3:** Clinical Global Impression (CGI) Scale among Drive-Mind 1 participants who attended all visits (N=170).

CGI-Severity at M0 Visit	N	(%)
Normal	0	(0)
Borderline mentally ill	0	(0)
Mildly ill	14	(8.3)
Moderately ill	123	(72.4)
Markedly ill	18	(10.7)
Severely ill	15	(8.9)
Among the most extremely ill patients	0	(0)
CGI-Improvement at M6 Visit, compared to M0*		
Improvement	127	(75.1)
No change or minimal change	40	(23.7)
Deterioration	1	(0.6)
NA	1	(0.6)
**CGI-Improvement at M12 visit, compared to M0**		
Improvement	134	(78.8)
No change or minimal change	34	(20.1)
Deterioration	2	(1.2)
NA	0	0
		

⁎ missing data for one subject at M6

### Treatment acceptance and adherence

Following clinical evaluations, all patients (n = 170) requiring psychiatric medication at M0 agreed to take it. Treatment acceptance was good, since only seven participants refused the prescribed medication (out of 157) at M6, and four refused it at M12 (out of 123).

Treatment adherence improved in the course of the study. At M6, 37% of the subjects reported having stopped the prescribed medication for non-medical reasons in the last 6 months. This proportion dropped to 26% at M12, after some of them resumed their medication.

### Stigma and quality-of-life

Self-report of feelings of shame at being diagnosed with a mental health problem decreased significantly between cohort initiation and the end of follow-up (55% *vs.* 17%, p<0.001).

The "anxiety" and "pain/discomfort" dimensions of the EQ5D5L (quality-of-life) were significantly improved (Table 4) as was the score for perceived health which significantly increased between M0 and M12 (66.49 vs. 72.99, p<0.001).

**Table 4:** Evolution of EQ-5D-5L scale scores among Drive-Mind 1 participants who attended all visits (N=170).

	Time point		p-value*
	M0	M12	
**Mobility (%)**			
I have no problems walking about	138 (81.2)	137 (80.6)	*0.203*
I have slight problems walking about	27 (15.9)	23 (13.6)	
I have moderate problems walking about	4 (2.4)	6 (3.6)	
I have severe problems walking about	0 (0.0)	4 (2.4)	
I am unable to walk about	0 (0.0)	0 (0.0)	
NA	1 (0.6)	0 (0.0)	
**Self-Care**			
I have no problems washing or dressing myself	156 (91.8)	149 (87.6)	*0.095*
I have slight problems washing or dressing myself	11 (6.5)	16 (9.5)	
I have moderate problems washing or dressing myself	2 (1.2)	2 (1.2)	
I have severe problems washing or dressing myself	0 (0.0)	2 (1.2)	
I am unable to wash or dress myself	0 (0.0)	1 (0.6)	
NA	1 (0.6)	0 (0.0)	
**Usual activities**			
I have no problems doing my usual activities	144 (84.7)	130 (76.5)	*0.243*
I have slight problems doing my usual activities	20 (11.8)	28 (16.6)	
I have moderate problems doing my usual activities	4 (2.4)	7 (4.1)	
I have severe problems doing my usual activities	1 (0.6)	3 (1.8)	
I am unable to do my usual activities	0 (0.0)	2 (1.2)	
NA	1 (0.6)	0 (0.0)	
**Pain/discomfort**			
I have no pain or discomfort	91 (53.5)	112 (65.9)	*0.004*
I have slight pain or discomfort	40 (23.7)	38 (22.5)	
I have moderate pain or discomfort	27 (16.0)	12 (7.1)	
I have severe pain or discomfort	10 (5.9)	8 (4.7)	
I have extreme pain or discomfort	1 (0.6)	0 (0.0)	
NA	1 (0.6)	0 (0.0)	
**Anxiety/depression (%)**			
I am not anxious or depressed	102 (60.0)	124 (72.9)	*0.003*
I am slightly anxious or depressed	32 (18.9)	29 (17.2)	
I am moderately anxious or depressed	20 (11.8)	9 (5.3)	
I am severely anxious or depressed	13 (7.7)	6 (3.6)	
I am extremely anxious or depressed	2 (1.2)	2 (1.2)	
NA	1 (0.6)	0 (0.0)	
**Quality of life (mean (sd))**			
Visual analogue scale	66.57 (17.35)	73.09 (16.78)	*<0.001*

⁎ Wilcoxon paired test for categorical variables, paired t-test for continuous variables

### Drug use and methadone

Results are shown in table 2. After 12 months of intervention, we observed a significant decrease in the number of subjects reporting heroin use in the past 6 months (61% vs. 69%, p= 0.045). On the other hand, the frequency of use only slightly decreased (median = 20 days vs. 22 days in the last 30 days, p=0.768). Regarding methamphetamine use, we observed a slight increase in the proportion of participants declaring regular use at M12, albeit not statistically significant (27% vs. 25%, p= 0.728). Consumption of alcohol, however, progressively and significantly decreased over the twelve months of intervention (26.5% at M0 vs 18.8% at M12, p=0.016). The proportion of subjects receiving methadone treatment did not significantly change between M0 and M12 (65% and 68%, respectively).

Lastly, we did not observe any HIV sero-conversion in the 12-month period of follow-up.

### Sensitivity analysis

A sensitivity analysis was performed to measure the impact of the intervention in a subgroup of subjects naïve to any psychiatric follow-up. Among the 233 subjects included in DRIVE-Mind, 72 subjects were included in the DRIVE-cohort in the 6 months prior to the start of DRIVE-Mind and did not undergo any type of psychiatric intervention. Results from this analysis are in line with the results from the main analysis for the effect of the psychiatric intervention. All eligible subjects in this subgroup accepted the intervention. Of these, 49 (68%) completed the follow-up. Significant improvement was observed for the two main psychiatric disorders studied and suicide risk. The CGI Scale analysis indicated a clear improvement in symptoms after one year of intervention for 38/49 participants (78%).

## Discussion

To our knowledge, this is one of the first prospective studies evaluating the impact of a psychiatric intervention, at community level among PWID in a LMIC.^22^ In this particularly exposed and highly stigmatized population suffering simultaneously from psychiatric and drug use disorders, the strategy proved efficient. The vast majority of participants requiring a psychiatric intervention agreed to be involved in the cohort and among them, 76% were still in follow-up after 12 months. More significantly, nearly 4/5 were clinically improved after 12 months, self-rated health was significantly improved and perceived stigma considerably reduced.

The magnitude of the benefit of the intervention, particularly for depression, is significant but consistent with the data in the literature. Only about 10 to 30% of subjects diagnosed with a major depressive disorder usually do not respond to antidepressant treatment.^41^^,^^42^ This benefit is also undoubtedly reinforced by the fact that we did not differentiate primary disorders from substance-induced disorders in our data analysis. The mere reduction in alcohol use and in numbers of people injecting heroin in our population sample could partly explain our results. The global support provided to all PWID recruited in the cohort, including administrative and peer support, linkage to HIV care and methadone treatment, participated probably also to the overall improvement of the psychiatric status of this population. And lastly, around half of the DRIVE-Mind project cohort had a previous psychiatric contact or follow-up and around 10% still reported being under treatment at DRIVE-Mind cohort initiation. This could have had an impact on the observed benefit of the intervention and could have participated in the good acceptance of the intervention. Nevertheless, the sensitivity analysis showed that among PWID naïve to any psychiatric follow-up, the benefit of the intervention was similar to that for the whole sample. On the other hand, the persistence of psychiatric disorders despite previous treatment could also mean that these patients were unresponsive to treatment or poorly adherent to care, and therefore more difficult to treat than patients naïve to any treatment.

Despite this success, the proportion and characteristics of participants lost to follow-up is nevertheless a concern. Around a quarter could not be reached at M12. However, this attrition rate is common in psychiatric populations, most commonly around 30%, but varying from 4 to 46% depending on the study setting, population, service type and definitions of involvement in care used.^41^^,^^43^, ^44^, ^45^, ^46^ Predictive factors for attrition in the literature are gender (male), marital status (single), younger age, low socioeconomic status, psychosocial problems, ethnicity, criminal history and substance abuse.^43^^,^^45^^,^^46^ Our study participants were mainly male, all of them were using drugs and those lost to follow-up were more socially deprived at baseline, less often on methadone and with lower efficacy of antiretroviral therapy for those infected with HIV. More than half of those missing at month 12 (excluding deceased participants) had been incarcerated or referred by authorities to a compulsory centre in the course of the one-year follow-up. Our data is in line with the literature and underlines the need to focus attention on subjects with comorbid disorders, socially impaired and involved in legal issues. Predictive factors for disengagement from care should been screened early and supportive environments proposed for this subpopulation.^47^ Providing free access to basic care such as methadone for opioid-dependent subjects at an early stage of care should be considered, as should close administrative support.

Among the participants still in follow-up after 12 months, drug use profiles did not significantly change following the psychiatric intervention except for alcohol consumption which decreased significantly and a slight reduction in the number of participants injecting heroin. In fact, many participants were already treated with methadone or had a previous contact with a psychiatrist before cohort initiation, drawing benefit from of these interventions before the cohort assessment. On the other hand, recruitment in the cohort was not associated with a commitment to quit drug use or to comply with treatment. The baseline depression values were comparable to other studies conducted among PWID in Vietnam but not all, probably because of the heterogeneity of the methodologies used.^12^^,^^48^ The dynamics of mental health improvement could also be independent from drug use behaviors, particularly if we consider that basic needs (harm reduction services, access to methadone treatment) were already met. Improvements in self-perceived health and stigma are probably much more related to mental health improvement than to any change in drug use profiles, which were virtually unchanged, except for alcohol. Nevertheless, a comprehensive, global approach using structured psychosocial interventions is still necessary, but faces several obstacles.^49^ Except for access to methadone treatment, “technically” fairly easy to implement, addiction medicine is very poorly developed in many parts of the world. More significantly, a comprehensive approach to comorbid disorders, involving a multidisciplinary staff and continuing training, appears often a luxury or a utopia given the many public health priorities in most low-middle and also high-income countries. That is why non-professional and peer-supported interventions are crucial in many places.^22^^,^^50^ Despite being initially uncomfortable in dealing with mental health issues, CBO staff involved in our project were finally at ease after structured training and creation of their own tools in assessing and referring participants, under supervision of psychiatrists.^35^ They participated in all steps of the project design and received a regular salary. Their intervention probably participated greatly to decreasing the stigma associated with being diagnosed with a psychiatric disorder, as they acquired better understanding of mental health issues and were more self-confident in addressing these questions with their peers. The alliance between psychiatrists and peers involved in the project was excellent, and this is a key component of a successful intervention.^50^ Regular supervision and feed-back from mental health professionals remains necessary, particularly for the management of the most severe cases.

Altogether, providing consultations in a familiar and friendly context with CBO support, different from the psychiatric department, making consultations and treatments free, and increasing CBO mental health literacy were probably the three key points of the intervention.

Many questions are still open. Providing official status for peer-support workers trained to deal with specific issues in the community is a real question and would help to acknowledge the competencies they develop and stabilize their situation. The city of Hai Phong started to develop peer-supported interventions many years ago with the involvement of the city health authorities, national NGOs and international agencies, targeting key and hard-to-reach populations.^33^^,^^51^ It clearly participated in the success of the intervention presented here and this raises the question of the reproducibility of this type of mobilization of actors in other provinces. Cost and cost-effectiveness of the intervention need to be compared with the classic model of care to develop advocacy toward health authorities. Clinical concerns also arise. What is the medium-to-long term evolution of the mental health problems diagnosed in this population, and particularly, what is the prognosis of the psychotic disorders diagnosed in a context of frequent methamphetamine use? Is there any benefit of this mental health intervention on risk-prone drug-related or sexual behaviours in this highly exposed population?

Some limitations to this work should be mentioned. First, the study participants all had access to harm reduction services and methadone treatment, and some of them to earlier psychiatric follow-up, before cohort initiation. This could have had an impact on the psychiatric intervention, limiting its benefit. Nevertheless, considering the clear and significant improvement in mental health status in our study population, this is not a major concern. Secondly, we had no control group to help disentangle the benefits of the psychiatric intervention. For ethical reason, it was impossible to leave participants suffering from psychiatric disorders without care, or to simply offer them referral to the classic psychiatric health system, knowing that in a preliminary study, none of those diagnosed with a psychiatric disorder agreed to visit the mental health department for treatment/follow-up.^30^ Stigma was assessed with only one question on perceived shame at being diagnosed with a psychiatric disorder. This is probably not sufficient to draft definitive conclusions on the impact of peer-supported interventions on stigma associated with psychiatric status and care, but it is coherent with the literature.^21^^,^^52^ Lastly, our study was conducted on a relatively homogeneous sample of people who use drugs, all opioid-dependent, most using methamphetamine and all living in the same area. The same study should be replicated in different populations and places.

## Conclusion

Our community-based psychiatric intervention proved its ability to involve and maintain in care most of PWID diagnosed with depression, psychotic disorder and/or suicide risk. Their clinical situation and quality-of-life were significantly improved, and perceived stigma was reduced.

Developing a CBO-supported intervention for people suffering from comorbid disorders in LMICs is feasible and seems efficient. It is probably even easier to develop among subjects with psychiatric disorders in the general population, with lower levels of addictive behaviours. Nevertheless, populations cumulating vulnerabilities probably draw more benefit from peer-supported interventions because of more frequent marginalization and greater stigma.

## Contributors

NN, DDJ, LM, and SML conceptualized the study. NN, DDJ, LM, SML, OKTH, HDT, DR, GHT and RV designed the questionnaires. LM and SML conceptualized the intervention. SML, LM, HDT, KPM, TNTTB, GHT, VVH, LNT, DR, CQ, HQD, DNQ and OKTH participated in the implementation of intervention, supervised the work with the Community Based Organizations and the data collection. PT and RV performed the data analysis with input from SML, LM and NN. SML, LM and PT drafted the manuscript, NN, DDJ, JPM and JF edited the manuscript. NN, DDJ, LM, HDT, KPM, JF, PT, JPM and DL provided critical revisions. All authors have reviewed and approved the final version of the paper for submission.

## Data sharing statement

The following data will be made available on request addressed to the corresponding author: de-identified participant data, data dictionary, study protocol, informed consent.

## Declaration of interests

The authors declare no conflict of interest.

## Appendix Supplementary materials

Image, application 1

Image, application 2
