# Supplementary material for: Assessment of a psychiatric intervention at community level for people who inject drugs in a low-middle income country: the DRIVE-Mind cohort study in Hai Phong, Viet Nam
Source: Lancet Reg Health West Pac. 2021 Dec 13;18:100337. doi: 10.1016/j.lanwpc.2021.100337 (PMC8669310; doi:10.1016/j.lanwpc.2021.100337)
Supplement: Supplementary file 2 [file mmc2.docx]

**Supplementary material for the following research article: “Assessment of a psychiatric intervention at community level for people who inject drugs in a low-middle income country: the DRIVE-Mind cohort study in Haiphong, Viet Nam.”**

Table of content

[**1)** **Further details about the DRIVE RDS surveys** 1](#_Toc86186489)

[**2)** **Further details on CBO training on mental health** 1](#_Toc86186490)

[**3)** **Quick psychiatric screening tool** 2](#_Toc86186491)

[**4)** **Chlorpromazine and Imipramine equivalences in the DRIVE-Mind project** 3](#_Toc86186492)

## **Further details about the DRIVE RDS surveys**

*The DRIVE project (*Drug use and Infections in ViEtnam)

Each survey began with approximately 20 “seeds,” selected by community-based organization (CBO) staff for diversity (age, gender, HIV status) and for their large social networks of PWID. Each seed first participated in all study procedures, and then was given three numbered coupons to recruit new participants. As new individuals joined in the study, they were then given numbered coupons to recruit additional new participants, and so on until the required sample size was reached.

Criteria for inclusion in the different RDS surveys were: age 18 or over, self-reported current drug injection, confirmed by presence of recent skin injection marks and positive urinalysis for heroin and/or methamphetamine (Nal Von Minden, Germany), residence in Hai Phong, and ability to provide informed consent.

In each of the RDS surveys, we invited participants to join an open cohort study with follow-up every six months. The objective of the intervention study was to assist participants in initiating methadone treatment, entering and adhering to ART, education on safer injection and sexual behaviours and administrative support (health insurance card).

## **Further details on CBO training on mental health**

Supplementary table 1: Further details on CBO training on mental health

| CBO Training on mental health | - definition of mental health, mental illness, - main symptoms of depression and psychosis, - links between drug/alcohol use and psychiatric disorders, - general information on the treatment of depression and psychosis, - main side-effects of the treatments, - expected benefits of these treatments and time to action, - importance of good observance and need to maintain treatment after immediate remission, - motivational interviewing, psychosocial intervention, drugs and drug interactions with mental health (limited number of CBO members). |
| --- | --- |

## **Quick psychiatric screening tool**

| **Quick psychiatric screening tool**  **1** **Over the past 2 weeks** have you been bothered by these problems? | | | | |  |
| --- | --- | --- | --- | --- | --- |
| 1.a | Feeling nervous, anxious, or on edge | |  Not at all (0)   Several days (1)   More than half the days (2)   Nearly every day (3) | |  |
| 1.b | Not being able to stop or control worrying | |  Not at all (0)   Several days (1)   More than half the days (2)   Nearly every day (3) | |  |
| 1.c | Feeling down, depressed, or hopeless | |  Not at all (0)   Several days (1)   More than half the days (2)   Nearly every day (3) | |  |
| 1.d | Little interest or pleasure in doing things | |  Not at all (0)   Several days (1)   More than half the days (2)   Nearly every day (3) | |  |
| 1. **Over the past 2 weeks**, has the thought of harming yourself occurred to you? | | |  No   Yes | |  |
| 1. **In the past,** have you ever attempted suicide? | | |  No   Yes   Don’t know/No answer | |  |
| 1. **Have you ever.**   (answers for each question) | | |  | |  |
| 4.a | | believed that people were spying on you, or that someone was plotting against you, or trying to hurt you? |  No   Yes   Don’t know/No answer |  |  |
| 4.b | | believed that someone was reading your mind or could hear your thoughts or that you could actually read or hear what another person was thinking? |  No   Yes   Don’t know/No answer |  |  |
| 4.c | | heard things other people could not hear, such as voices? |  No   Yes   Don’t know/No answer |  |  |

## **Chlorpromazine and Imipramine equivalences in the DRIVE-Mind project**

Supplementary table 2: Chlorpromazine and Imipramine equivalences

| n=233 | **Risperidone** | | **Olanzapine** | | **Sulpriride** | | **Mirtazapine** | | **Sertraline** | |
| --- | --- | --- | --- | --- | --- | --- | --- | --- | --- | --- |
| visit | **M0** | **M12** | **M0** | **M12** | **M0** | **M12** | **M0** | **M12** | **M0** | **M12** |
| **Number of patients** | 60 | 30 | 80 | 53 | 83 | 22 | 109 | 41 | 95 | 43 |
| **Dosage (min-max) mg/day** | 1-4 | 1-6 | 5-20 | 5-20 | 50-100 | 50-150 | 30 | 15-60 | 50-100 | 50-200 |
| **Mean dosage (SD) mg/day** | 2.12 (0.52) | 2.5 (1.07) | 7.25 (11.96) | 8.11 (4,52) | 75.90  (25.14) | 75.00 (29.88) | 30  (0) | 29.27 (6.67) | 68.42 (24.25) | 82.56 (36.00) |
| **Equivalent (mg/day)**  **-chlorpromazine^1^**  **-imipramine^2^** | 106^1^ | 125^1^ | 145^1^ | 162^1^ |  |  | 81^2^ | 79^2^ | 95^2^ | 115^2^ |
